# Solute Diffusion in Styrenic Triblock Copolymer Organogels

**DOI:** 10.1021/acsapm.6c01038

**Published:** 2026-05-15

**Authors:** Kenneth P. Mineart, Nicholas G. DeVita, Ridwana Bashar

**Affiliations:** Department of Chemical Engineering, 4517 Bucknell University, Lewisburg, Pennsylvania 17837, United States

**Keywords:** organogels, block copolymer, solute transport, structure–property relationships, SAXS

## Abstract

Gels composed of styrenic triblock copolymer and aliphatic solvent have been recently considered for use in transdermal drug delivery systems (TDDSs) due in part to their adhesive properties and nonpolar chemistry. Past research conducted on these block copolymer organogels has provided some formulation strategies to optimize their adhesiveness, drug retention, in vitro drug release, and in vivo delivery performance. However, the aforementioned studies’ characterization of extrinsic properties and use of empirical modeling limit the generalizability of their outlined strategies. The current study presents solute diffusivity–an intrinsic material property–for various copolymer organogels including those with different copolymer molecular weights and concentrations, as well as several unique solutes. Furthermore, the gathered data sets are fitted with a theoretical model that accounts for the nanostructure of organogels including their impenetrable discrete domains (polystyrene) and continuous phase comprising solvated midblocks (poly­[ethylene-propylene] or poly­[ethylene-butylene]) and gel solvent. This model yields the hydrodynamic radius of the penetrant (i.e., solute molecules or aggregates) and its average rate of movement through a given gel. Interpretation of diffusivity values and model fit parameters suggests that copolymer concentration has a considerably stronger effect on solute diffusion than copolymer molecular weight. For example, increasing concentration from 5 wt% to 20 wt% yields a 50% reduction in solute diffusivity whereas molecular weight (at a fixed concentration) is observed to have a negligible impact. Our findings also highlight that solute aggregation behavior must be carefully considered when designing organogel-based TDDSs. The hydrodynamic radii of solutes employed do not correlate with their molecular weights (OA: 282.5 g/mol, 0.3 nm; SMO: 428.6 g/mol, 0.7 nm; AOT: 444.6 g/mol, 2.6 nm; STO: 957.5 g/mol, 0.8 nm), which stems from differing self-assembly behavior in the gel solvent. We anticipate that the presented results and analysis will enable more effective bottom-up design of block copolymer organogels for TDDSs.

## Graphical abstract

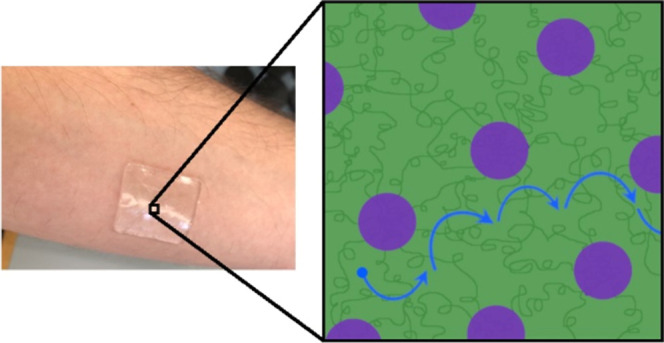

## Introduction

Polymer gels are excellent candidates for drug delivery media because of their widely tunable chemistries and properties.^1^ Transdermal drug delivery systems (TDDSs) are one such type of media that are used to transmit desired compounds into the body through the skin.^2^ The transdermal approach has several practical benefits including increased convenience, decreased invasiveness, and the ability to deliver therapeutic compounds locally (i.e., at the site of application) in addition to into the bloodstream.^3^ As a result, there are several TDDS-based products available including both over-the-counter (e.g., smoking cessation and pain management) and specialized (e.g., breast and skin cancer therapy) offerings.^4,5^ TDDSs must exhibit several attributes to be effective including holding and metering desired pharmaceutical/therapeutic compounds and adhering to and elastically deforming with the skin at the application site. The latter criteria has led to investigation of several biocompatible pressure-sensitive adhesives as at least one component of TDDSs.^6^

Materials comprising styrenic triblock copolymers (also referred to as thermoplastic elastomers^7^) have been established as pressure-sensitive adhesives for some time,^8^ but have only recently been considered as a component in TDDSs. Alongside copolymer, organogels formulated for TDDSs contain an aliphatic gel solvent that might include mineral oils, aliphatic plasticizers, and/or hydrocarbon tackifying resins.^9–12^ The combination of styrenic triblock copolymer, which must have an aliphatic hydrocarbon midblock in this case, and aliphatic solvent results in a nanostructure consisting of discrete, nearly pure polystyrene domains (commonly spherical) within an aliphatic (i.e., midblock/solvent-rich) matrix phase.^13,14^ Furthermore, the matrix phase is a semidilute solution of copolymer midblocks each of which is bound at both ends to polystyrene domains. The key characteristic that causes this nanostructure to form is the incompatibility between styrenic endblocks and the aliphatic character of its midblock and the gel solvent, as well as the fact that the endblock concentration is relatively low (≈1–15 wt%).^14^ Analogous hydrogels can be synthesized based on triblock copolymers containing hydrophobic endblocks and hydrophilic midblocks like poly­(ethylene glycol) or polymethacrylate derivatives,^15–17^ but their large-scale production is not yet as developed as the aliphatic versions considered here.

Previous work has shown that styrenic triblock copolymer organogels can be formulated to match the performance of commercially available testosterone^9^ and ibuprofen^12^ transdermal patches. Furthermore, the studies identify several impactful formulation parameters: (1) a larger proportion of tackifying resin in the aliphatic solvent increases total drug release,^9^ (2) a larger proportion of tackifying resin in the aliphatic solvent, as well as higher copolymer concentration, increases patch adhesiveness,^9^ and (3) lower molecular weight copolymer increases drug release rate and cumulative amount released.^12^ The aforementioned conclusions were made through design of experiments factorial analysis, and no attempt was made to relate them to foundational transport theory or polymer physics. We have recently taken several steps to bridge this knowledge gap starting with development of a method that enables determination of solute diffusivity through styrenic triblock copolymer gels.^18^ The current paper presents solute diffusivity values for styrenic triblock copolymer gels composed of different copolymer concentrations, multiple copolymer molecular weights, and a variety of solutes. We interpret this diverse data set using a single, nanostructure-based model enabling underlying physics to be extracted. The collective results and analysis presented herein provide a strong basis for the well-informed, bottom-up design of TDDSs using styrenic triblock copolymer organogels.

## Experimental Section

### Materials

Gels examined in this study are composed of a styrenic triblock copolymer (poly­[styrene-*b*-(ethylene-*co*-butylene)-*b*-styrene] (SEBS) or poly­[styrene-*b*-(ethylene-*co*-propylene)-*b*-styrene] (SEPS)) and mineral oil. The molecular attributes of triblock copolymers, which were provided by Kraton Polymers (SEBS) and Kurrary Inc. (SEPS), are summarized in Table 1. Mineral oil (MO) was provided by Sonneborn (Hydrobrite 200 PO) and consists of a mixture of oligomeric aliphatic and alicyclic hydrocarbons. The dynamic viscosity of this mineral oil grade at 21 °C was found to be (101.4 ± 0.2) mPa-s using a Brookfield (Middleborough, MA, USA) DVE viscometer with small sample adapter (6.7 mL) and jacketed temperature-control system. Dioctyl sulfosuccinate sodium salt (AOT), oleic acid (OA), sorbitan monooleate (SMO), sorbitan trioleate (STO), and poly­[hydroxystearic acid] (pHSA) were incorporated into gels as diffusion probes (Figure 1). AOT (≥97%), SMO (Span 80) and STO (Span 85) were purchased from Sigma-Aldrich, and OA (≥99%) was purchased from TCI America. pHSA was provided by Lubrizol Corp (Solsperse 3000). Finally, toluene (≥99.5%, VWR) was used in the gel preparation process.

**Table 1:** Characteristics of Triblock Copolymers Used in the Current Study: Weight-Averaged Molecular Weight, *M*_*w*_, Polystyrene Mass Fraction, *f*_*S*_, and Ethylene, Propylene, and Butylene Compositions within Each Copolymer Midblock

polymer	*M*_*w*_ (kg/mol)^a^	*f*_*S*_ (wt%)^b^	midblock composition (wt%)^b^ ^,^ ^c^		
			ethylene	propylene	Butylene
S_10_EB_55_S_10_	75.9 ± 2.6	27.5	59.1	-	40.9
S_22_EB_114_S_22_	157.5 ± 7.9	27.8	56.8	-	43.2
S_32_EB_170_S_32_	234.9 ± 8.1	30.9	59.3	-	40.7
S_61_EP_298_S_61_	421.0 ± 18.0	29.1	56.9	43.1	-

a *M*_*w*_ values were determined via static light scattering measurements.

b *f*_*i*_ values were determined via H^1^–NMR where *f*_*i*_ is *f*_*S*_, *f*_ethylene_, *f*_propylene_, or *f*_butylene_.

c Midblock composition was determined by *f*_*j*_/(*f*_ethylene_ + *f*_propylene_ + *f*_butylene_) where *f*_*j*_ is *f*_ethylene_, *f*_propylene_, or *f*_butylene_.

**Figure 1:**
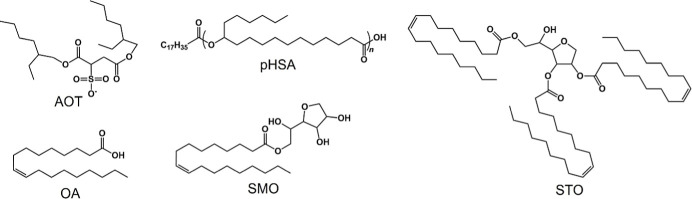
Chemical structures for diffusion probes (i.e., solutes) used in the present study: dioctyl sulfosuccinate sodium salt (AOT), oleic acid (OA), poly­[hydroxystearic acid] (pHSA), sorbitan monooleate (SMO), and sorbitan trioleate (STO).

Gels were prepared by dissolving the desired amounts of each component in toluene at a 20:1 toluene volume to gel mass ratio. For example, 0.05 g STO, 1.00 g S_22_EB_114_S_22_, and 3.95 g MO were dissolved in 100 mL of toluene. Solutes were added at a concentration of either 0.5 wt% (OA), 1.0 wt% (AOT, STO, pHSA), or 1.5 wt% (SMO) based on experimental sensitivity (FTIR). Copolymer concentration varied systematically. Upon full dissolution of gel components into the toluene solution, toluene was removed via rotary evaporator. The resultant gels were annealed in a vacuum oven at 140 °C and 0.05 atm for 18–24 h. Then, gels were pressed into disks (ca. 1.65 mm (thickness) × 25 mm (diameter)) on a Carver melt press operated at 100–180 °C (depending on copolymer molecular weight and concentration) and minimal applied pressure.

Gel disks were swelled after annealing but prior to analysis such that no further swelling would occur during characterization (namely, diffusion experiments). Swelling was conducted in a gel solvent-matched solution so that diffusion probe concentration was mostly maintained. For example, disks composed of 0.05 g STO, 1.00 g S_22_EB_114_S_22_, and 3.95 g MO were submersed in a 1.0 wt% STO/99.0 wt% MO solution (e.g., 0.35 g SMO/34.65 g MO). Swelling was conducted at ambient temperature until gels achieved an equilibrium state as ascertained by periodic mass tracking. The time required to reach equilibrium and the final extent of swelling, *Q* (define by *m*_*f*_/*m*_*0*_ where *m*_*f*_ and *m*_*0*_ are the final and initial mass), varied depending on copolymer identity and concentration. All analysis below only considers the final copolymer concentration, *w*_*ABA*_ (in wt%), which was determined by accounting for the initial concentration, *w*_*ABA,0*_, and *Q* via *w*_*ABA*_ = *w*_*ABA,0*_/*Q*. Values of *w*_*ABA*_ for all gels, based on *w*_*ABA,0*_, are displayed in Tables S1 and S2.

#### Diffusion Experiments

Diffusion experiments followed the procedure in our previous publication.^18^ In short, experiments were conducted by submersing gel disks into pure MO and subsequently collecting FTIR spectra of each disk at regular intervals. The ratio of gel mass to supernatant MO volume was maintained at ca. 5:100 (g/mL) throughout all experiments, and gel mass was measured prior to each FTIR measurement to ensure negligible sample swelling occurred. During submersion, sample jars containing gel disks and supernatant MO were agitated by a shaker table maintained at 200 rpm and held at a fixed temperature (21 °C) using a controlled-temperature water bath. FTIR spectra were collected on a Thermo Scientific Nicolet iS10 spectrometer (0.5 cm^–1^ resolution and 32 scans/specimen) that was maintained at ambient temperature (ca. 21 °C) and purged with N_2_. Gels’ surfaces were wiped off prior to each FTIR measurement. Gel thickness was recorded at the beginning and end of diffusion trials (Tables S3 and S4); negligible change was observed over the duration of experiments.

#### SAXS Measurements

Small angle X-ray scattering (SAXS) measurements were conducted either on beamline 12-ID-B at the Advanced Photon Source (APS) within Argonne National Laboratory or on a Xeuss Xenocs 2.0 HR lab-source instrument. Experiments at the APS used 13.3 keV X-ray radiation (λ = 0.93 Å) and a Pilatus 2 M detector. Those on the Xeuss instrument used a microfocus sealed copper tube (λ = 1.54 Å) and a Pilatus3 R200 K detector. Sample-to-detector distances were 2.01 m (APS) or 2.52 m/0.57 m (Xeuss) and the 0.57 m position on the Xeuss utilized three vertical virtual detector positions. Exposure times were 1.0 s (APS) or 10 min at each configuration and detector position (Xeuss). Measurements were performed at ambient temperature and pressure (APS) or ambient temperature and under high vacuum (Xeuss).

2D SAXS intensity maps were converted into 1D profiles via azimuthal integration where the scattering vector magnitude, *q*, is related to the scattering half angle, θ, by *q* = 4πsin­(θ)/λ. Data collected at the two unique detector positions on the Xeuss were merged by vertically shifting data collected at 0.57 m by a constant factor to minimize differences in overlapping regions. The block copolymer and solute aggregate structure in gels was isolated in scattering profiles by subtracting thickness-corrected, amorphous MO scattering from gel scattering, *I*_gel_(*q*). Pure MO scattering was determined by subtracting empty capillary data, I_cap_(*q*), from MO-filled capillary data, I_MO+cap_(*q*). This is collectively captured by

$$I\left(q\right)=I_{gel}\left(q\right)-\frac{2L}{d_{cap}}\left(I_{MO+cap}\left(q\right)-I_{cap}\left(q\right)\right)$$

where *I*(*q*) is the scattering intensity arising from block copolymer and solute aggregate structure, 2*L* is the gel thickness (1.65–2.50 mm), and *d*_cap_ is the capillary inner diameter (1.98 mm).

## Results and Discussion

### Solute Diffusivity in Organogels

The diffusion experiments used in the present study take advantage of a solute concentration gradient between organogels and the surrounding MO, which translates to passive solute diffusion from the former into the latter. This process can be tracked by collecting FTIR spectra of gels over time since the concentration of each solute is directly related to the peak absorbance corresponding to their ester and/or acid functional groups (1710–1740 cm^–1^).^18^ The peak absorbance in this range, which is almost completely isolated from copolymer and MO peaks, decreases over submersion time for all gels considered as would be expected for a material releasing solute (Figure S1). Absorbance values can be subsequently converted into relative solute concentrations, *ĉ*, through comparison with the beginning of the diffusion experiments (i.e., *ĉ* = *c*_*t*_/*c*_0_ = *A*_*t*_/*A*_0_ where *c*_*t*_ and *c*_0_ are probe concentration at time *t* and 0, respectively, and *A*_*t*_ and *A*_0_ are ester/acid absorbance at time *t* and 0, respectively). Finally, the time-resolved, relative solute concentration data enables construction of a solute retention curve for any given organogel (Figures 2a and S2).

**Figure 2:**
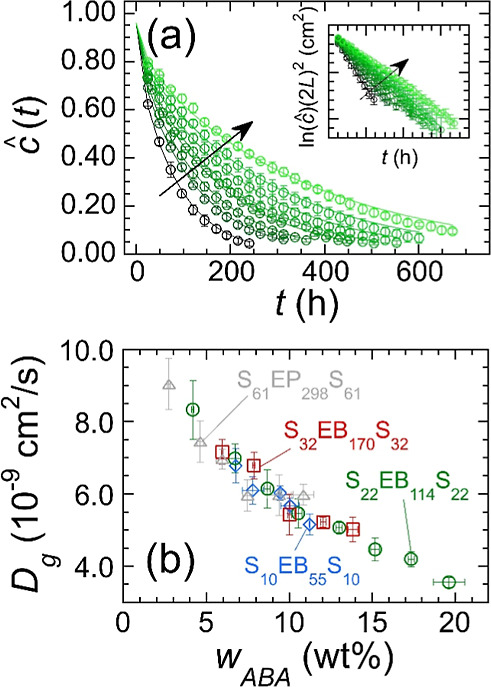
(a) Solute retention curves for AOT diffusion through gels composed of S_22_EB_114_S_22_ copolymer at increasing concentration from 4.2 wt% to 19.6 wt% (indicated by arrows). The inset shows data plotted in quasi-linearized form. Solid lines reflect fitting with a truncated form of eq 2: main figure = five terms, inset = first term only. (b) Solute diffusivity through organogels formulated with different copolymer concentrations and molecular weights (labeled).

In order to extract solute diffusivity from a retention curve, we must consider Fick’s second law in the context of the present experiments. Assuming that diffusion is negligible in the angular and radial directions (due to compositional homogeneity in the gels initially and the thin disk geometry, respectively), the second law can be simplified to

1 $$\frac{\partial ĉ}{\partial t}=D_{g}\frac{\partial^{2}ĉ}{\partial z^{2}}$$

where *t* is time, *D*_*g*_ is solute diffusivity through the gel, and *z* is position in the thickness direction (where *z* = 0 is defined as the center of the gel and *z* = *L* is the gel surface). Our experimental design further leads to identification of necessary initial and boundary conditions including (1) the initial relative solute concentration is unity everywhere within the gel (*ĉ*(0, *z*) = 1), (2) the solute concentration is negligible in the supernatant MO throughout the experiment (*ĉ*(*t*, *L*) ≈ 0), and (3) diffusion occurs from the center of the gel outward (∂*ĉ*(*t*, 0)/∂*z* = 0). Because our supernatant is not a true infinite sink, the second condition is only approximate and our solute diffusivities may be slight underestimates as a result. Solving eq 1 with these conditions and integrating across the full gel thickness (i.e., 2*L*) yields

2 $$ĉ\left(t\right)=\sum_{n=1}^{\infty }\frac{8}{{\left(2n-1\right)}^{2}\pi^{2}}\exp\left(\frac{-D_{g}\pi^{2}{\left(2n-1\right)}^{2}}{{\left(2L\right)}^{2}}t\right)$$

where the only fitting parameter is *D*_*g*_. While we use the first five terms of the infinite sum in eq 2 to fit data and extract solute diffusivities for subsequent consideration, qualitative trends can easily be ascertained (see insets in Figures 2a and S2) by considering the first term alone which indicates that the slope of ln­(*ĉ*)­(2*L*)^2^ vs *t* is proportional to -*D*_*g*_ at moderate to long time scales (in our case, *t* > 50 h). In all cases, the model captures solute retention curves well providing confidence in the diffusivity values extracted from experiments. Solute retention curves (Figure S2) and extracted diffusivity values (Figure 2b) indicate that copolymer concentration has a significant impact on solute diffusion – increasing copolymer concentration leads to decreasing rate of diffusion. Alternatively, copolymer molecular weight has little to no observable effect. It is also worth noting that the extracted solute diffusivity values (Figure 2b) all fall 1–2 orders of magnitude lower than others’ explorations of comparably sized solutes in hydrogels (∼10^–9^ cm^2^/s compared to ∼ 10^–7^-10^–8^ cm^2^/s).^19–21^ This reduction is not surprising given that the MO used in the present study has a viscosity ∼ 10^2^ times greater than water at ambient temperature.

### Nanostructure-Based Model

Before proceeding with interpretation of solute diffusivity values, the relationship between nanoscale structure and solute diffusivity in organogels must be carefully considered. Among several available theories that describe diffusion through polymeric gels, Petit et al.^19^ have developed a model that relates macroscopic solute diffusion to the nanoscopic hopping of solute molecules from gap to adjacent gap in a gel network paired with the diffusion of solute through solvent via

3 $$\frac{1}{D_{g}^{\prime }}=\frac{1}{D_{0}}+\frac{1}{k\xi^{2}}$$

where *D*_*g*_ *’* and *D*_*0*_ are solute diffusivity through a homogeneous gel and pure solvent, respectively, *k* is hopping frequency (i.e., the rate at which solutes hop between gaps in the polymer network), and ξ is the gel mesh size (i.e., the distance between gaps in a polymer network, or semidilute solution–see Figure 3). Solute diffusivity can be further converted to hydrodynamic radius of the solute, *r*_*h*_, using the Stokes–Einstein relationship

4 $$r_{h}=\frac{k_{B}T}{6\pi \mu D_{0}}$$

where *k*_*B*_ is Boltzmann’s constant, *T* is absolute temperature, and μ is solvent viscosity.

**Figure 3:**
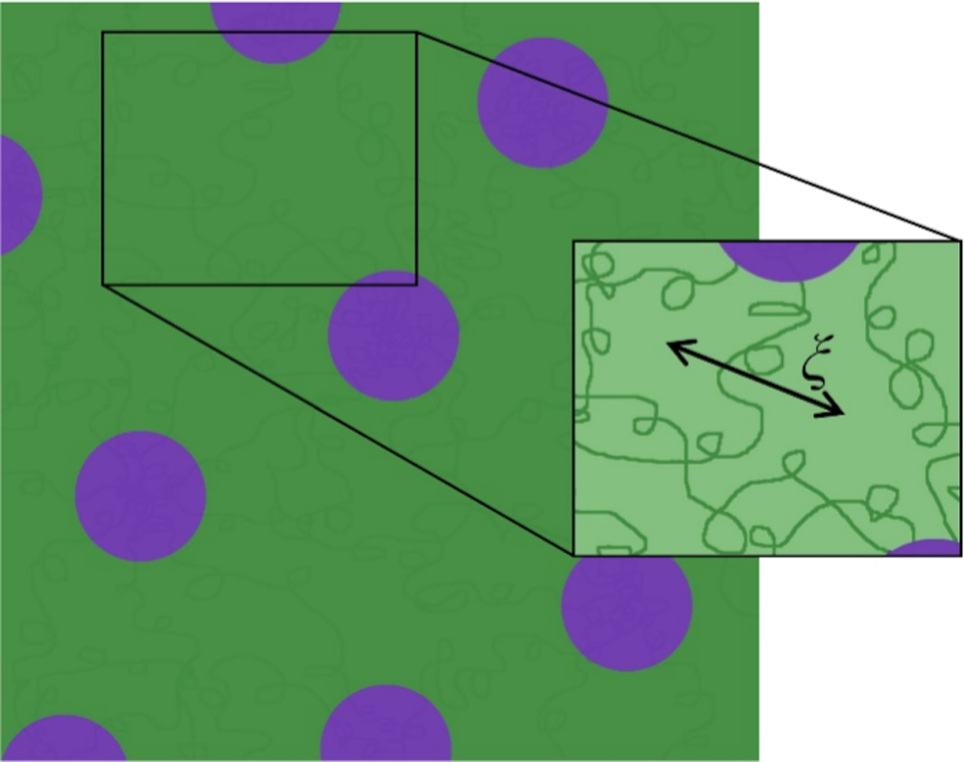
Schematic representation of a styrenic triblock copolymer gel’s nanoscale structure including spherical (purple) endblock domains and a continuous (green) matrix phase. The inset highlights the theoretical arrangement of midblocks within the matrix phase and indicates the mesh size parameter (ξ). Polystyrene domain volume fraction (ϕ_*S*_) is reflected by the fraction of space occupied by the spherical (purple) endblock domains.

As mentioned above, eq 3 describes solute diffusion through a homogeneous gel, which is one that is relatively uniform in its spatial distribution of solvent and polymer chains so that the two terms in eq 3 are valid everywhere within the gel. The nanoscale structure of styrenic triblock copolymer gels is not homogeneous in this sense due to the solute-impenetrable, endblock domains (Figure 3). Others^22^ have found success in using the obstruction theory of Mackie-Meares^23^ to model ion diffusion in similar materials

5 $$\frac{D_{g}}{D_{g}^{\prime }}={\left(\frac{1-\phi_{S}}{1+\phi_{S}}\right)}^{2}\equiv \psi$$

where ϕ_*S*_ is the volume fraction of the styrenic endblock domains in the organogel. For brevity, we use ψ to reflect this ‘correction factor’ for gel heterogeneity moving forward. The combination of eqs 3 and 5 provide an overall relationship between solute diffusivity and features of a given styrenic triblock copolymer organogel

6 $$\frac{\psi }{D_{g}}=\frac{1}{D_{0}}+\frac{1}{k}\left(\frac{1}{\xi^{2}}\right)$$

Equation 6 will be used to model solute diffusivity data across a variety of gel formulations where *D*_*0*_ and *k* are used as fitting parameters and the two structural features can be defined for each organogel formulation: ψ (through its relationship to ϕ_*S*_) and ξ.

Small-angle X-ray scattering (SAXS) has been used extensively to acquire domain geometry information on triblock copolymer gels including ϕ_*S*_.^24^ In line with the anticipated structure of our organogels (Figure 3), the copolymer contribution to 1D SAXS patterns is fitted with a spherical form factor and a structure factor that reflects liquid-like sphere ordering. The spherical form factor, *P*(*q*), is mathematically represented by the expression

7 $$P\left(q\right)={\left(\frac{\sin\left(qr_{S}\right)-qr_{S}\cos\left(qr_{S}\right)}{{\left(qr_{S}\right)}^{3}}\right)}^{2}$$

where *r*_*S*_ is the radius of the styrenic endblock domains. The structure factor, *S*(*q*), for liquid-like ordering of these spheres is captured by the expression

8 $$S\left(q\right)=1+4\pi \left(\frac{\phi_{hs}}{4\pi r_{hs}^{3}/3}\right)\int_{0}^{\infty }\left(g\left(r\right)-1\right)\left(\frac{\sin\left(qr\right)}{qr}\right)r^{2}dr$$

where ϕ_hs_ and *r*_*hs*_ are the volume fraction and radius, respectively, of theoretical hard spheres that define the separation of styrenic endblock domains (i.e., the center-to-center distance between two adjacent domains is defined by 2*r*_*hs*_ on average) and *g*(*r*) is their radial distribution function. This model, which is collectively reflected by

9 I(q)=αP(q)S(q)+agg+bkg

where α is a weighting factor, agg is scattering from noncopolymer aggregates (discussed more below), and bkg is incoherent background scattering, fits data well for all gels (Figures S3 and S4). (A detailed analysis can be found in our separate publication focused on the structure of these materials,^14^ and fitted parameters are provided in Tables S5–S10.) Applying some geometric intuition further enables the fitted model parameters to be converted into the endblock domain volume fraction via ϕ_*S*_ = ϕ_hs_(*r*_*S*_/*r*_*hs*_).^3^ The endblock domain volume fraction can be calculated separately using formulation quantities and molecular characteristics: ϕ_*S*_ = *f*_*S*_ *w*_*ABA*_(ρ_gel_/ρ_S_) where ρ_*S*_ and ρ_gel_ are the densities of polystyrene and of a given gel, respectively. There is excellent agreement between values ascertained from SAXS analysis and those calculated—experimental values are within 10% of theoretical expectation (Figure 4a). Small differences likely stem from measurement uncertainty during sample formulation. Therefore, we use the ϕ_*S*_ values acquired by SAXS for all subsequent analysis. Corresponding values of ψ, which decreases with increasing ϕ_*S*_, range from 0.97 to 0.84 (Figure 4a and Tables S9 and S10).

**Figure 4:**
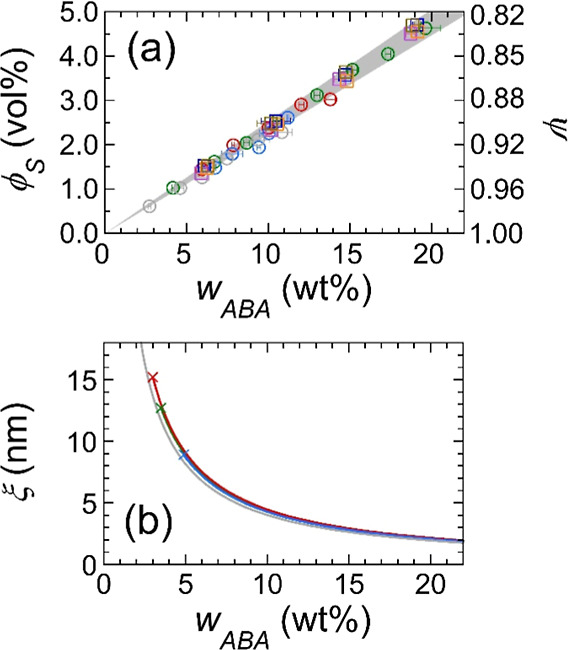
(a) Polystyrene endblock domain volume fraction with corresponding ψ “correction” and (b) matrix domain mesh size for all formulated organogels. In (a), data represented by circles contain AOT as the solute and vary in copolymer identity—blue: S_10_EB_55_S_10_, green: S_22_EB_114_S_22_, red: S_32_EB_170_S_32_, gray: S_61_EP_298_S_61_—whereas data represented by squares contain S_22_EB_114_S_22_ as the copolymer and vary in solute identity—pink: OA, orange: pHSA, navy: SMO, gold: STO. (Tabulated data can be found in Tables S9 and S10.). The gray region defines the theoretically anticipated range of values. In (b), lines correspond to eq 10 for each copolymer (solute identity has no effect)—blue: S_10_EB_55_S_10_, green: S_22_EB_114_S_22_, red: S_32_EB_170_S_32_, gray: S_61_EP_298_S_61_—and the *x* at the left end point of each reflects c*.

Conversely, there is not an experimental method to measure ξ in these materials with enough confidence to support the current work; we must rely solely upon theory to acquire these values. Scaling theory developed by de Gennes^25^ has generally been accepted as a good approximation for mesh size in semidilute polymer solutions

10 $$\xi =R_{g}{\left(\frac{c}{c^{*}}\right)}^{-\nu }$$

where *R*_*g*_ is the radius of gyration, *c* is the actual polymer concentration, c* is the polymer overlap concentration, and ν is a scaling exponent that depends on the solvent-polymer compatibility. We expect that mineral oil acts close to a theta solvent for poly­(ethylene-butylene) and poly­(ethylene-propylene) midblocks and, therefore, conduct subsequent analysis with ν = 1. As a reminder, we are assessing ξ of the copolymer midblocks in the matrix phase of organogels. This means that *R*_*g*_ is that of the copolymer midblock and can be determined by

11 $$R_{g}=\sqrt{C_{\infty }Nl^{2}}$$

where C_∞_ is the Flory characteristic ratio (6.4–6.5 for our copolymers’ midblocks), *N* is the number of bonds along the midblock backbone, and *l* is the length of a carbon–carbon bond. Equation 11 yields *R*_*g*_ values of 8.9, 12.7, 15.3, and 21.7 nm for the midblocks of S_10_EB_55_S_10_, S_22_EB_114_S_22_, S_32_EB_170_S_32_, and S_61_EP_298_S_61_ copolymers, respectively. The polymer (i.e., midblock) concentration in the matrix phase is found based on formulation parameters

12 $$c=\frac{\left(1-f_{S}\right)w_{ABA}}{\rho_{m}\left(1-f_{S}w_{ABA}\right)}$$

where ρ_*m*_ is the density of the matrix phase (0.865 g/cm^3^). Finally, c* of the midblock in the matrix phase is determined by

13 $$c^{*}=\frac{M_{w,B}}{4/3\pi R_{g}^{3}}$$

where *M*_*w,B*_ is the midblock molecular weight. eq 13 yields c* values of 0.031 g/cm^3^, 0.022 g/cm^3^, 0.018 g/cm^3^, and 0.012 g/cm^3^ for the midblocks of S_10_EB_55_S_10_, S_22_EB_114_S_22_, S_32_EB_170_S_32_, and S_61_EP_298_S_61_ copolymers, respectively. The resultant relationships highlight that copolymer concentration has a strong influence on mesh size whereas the impact of copolymer molecular weight is insignificant (Figure 4b).

### Role of Copolymer Factors

In terms of triblock copolymer factors, this study is focused on the impact of concentration and molecular weight on solute diffusion through organogels. Note, the four unique copolymers used in this study range in total molecular weight from 76,000 g/mol to 421,000 g/mol, but have relatively unchanged endblock fractions, *f*_*S*_ ≈ 27.5–30.9 wt% (Table 1). Linear regression of ψ-corrected diffusivity data to the form of eq 6 (i.e., plotting ψ/*D*_*g*_ vs 1/ξ ^2^) was used to yield 1/*D*_*0*_ (intercept) and 1/*k* (slope) (Figure 5). Extracted values for both *D*_*0*_ and *k* fall within experimental uncertainty across the copolymer molecular weights examined (Table 2). It is anticipated that *D*_*0*_ will remain constant since it is, by definition, solute diffusivity in the absence of copolymer. The observation that *k* does not vary means that the impact of copolymer factors is constrained to the structural features of gels. As a reminder, concentration has a strong influence on mesh size and the heterogeneous correction factor whereas molecular weight does not affect either structural feature (when ν = 1). Since neither parameter varied significantly, we collectively fit diffusivity data across all concentrations and molecular weights to determine overall values: *D*_*0*_ = (7.95 ± 0.63)×10^–9^ cm^2^/s and *k* = (1.89 ± 1.01)­x10 ^5^ s^–1^. Further interpretation of the former translates to an AOT hydrodynamic radius of 2.7 ± 0.2 nm (eq 4), which is in moderate agreement with others’ attempts^26–28^ to quantify this parameter for AOT reverse micelles (more on this below).

**Figure 5:**
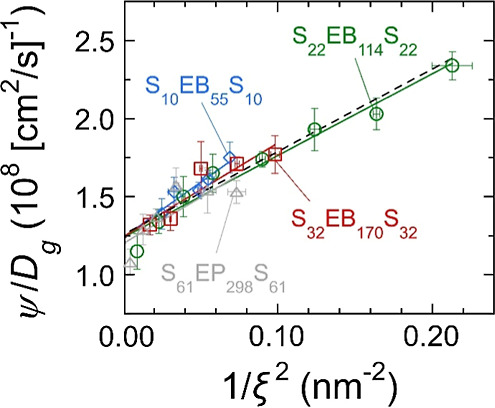
Solute diffusivity values for AOT diffusion through organogels formulated with different copolymer concentrations and molecular weights (labeled) presented in the linearized form of eq 6. Solid lines are linear fits to each copolymer series, and the dashed line is an overall fit to all data.

**Table 2:** Parameter Values Extracted from Fitting Solute Diffusivity Data in Figure 5 for Different Copolymer Identities Along with Resultant Values from Fitting all Data Together

copolymer	*D*_*0*_ (10^–9^ cm^2^/s)	*k* (10^5^ s^–1^)
S_10_EB_55_S_10_	8.06 ± 0.67	1.43 ± 0.51
S_22_EB_114_S_22_	8.06 ± 0.74	1.91 ± 0.25
S_32_EB_170_S_32_	8.03 ± 0.84	1.69 ± 0.87
S_61_EP_298_S_61_	8.34 ± 0.90	1.71 ± 1.07
all data	7.95 ± 0.63	1.89 ± 1.01

Others’ work^12^ found that the rates of solute release into aqueous medium and solute permeation from gels into and through skin decreased with increasing copolymer molecular weight. Their gels contained copolymers with similar composition as the present systems (*f*_*S*_ ≈ 30 wt%), but lower molecular weights (45,000–176,000 g/mol). In comparison, the present data shows little to no dependence on molecular weight. This discrepancy is most likely linked to the molecular weight range examined. In particular, the cited work utilized copolymers with polystyrene endblocks (ca. 7000–26,000 g/mol) that span across the entanglement molecular weight of polystyrene (*M*_*e,PS*_ ≈ 13,000 g/mol^29^) whereas endblocks in the present gels are near or above *M*_*e,PS*_. For cases where endblocks are well-below *M*_*e,PS*_, copolymer chains exhibit considerably faster endblock hopping and translational diffusion^30^ potentially leading to increased solute hopping frequency. Additionally, midblock bridging between polystyrene domains is likely lower in gels with smaller copolymers,^31^ which could result in greater heterogeneity in the distribution of copolymer molecules^13^ and consequently the presence of less obstructed pathways for solute diffusion.

#### Role of Solute Identity

To study diffusion in the present systems further, organogels were prepared with one of four alternative solutes: oleic acid (OA, 282.5 g/mol), sorbitan monooleate (SMO, 428.7 g/mol), sorbitan trioleate (STO, 957.7 g/mol), or poly­[hydroxystearic acid] (pHSA, molecular weight unknown). It is worth noting that these are model drug compounds not active pharmaceutical ingredients. Like AOT, they were specifically chosen because they contain acid and/or ester functionality enabling clear resolution in FTIR spectra and due to their capacity to be dissolved/dispersed in aliphatic solvent. The copolymer S_22_EB_114_S_22_ was used in all of these gel formulations for consistency. Furthermore, gels composed of each solute were prepared at four copolymer concentrations (*w*_*ABA*_ ≈ 6 wt%, ≈11 wt%, ≈15 wt%, and ≈19 wt%). Solute retention data for these 16 unique organogel formulations (Figure S5) are well-described by the model derived above (eq 2) enabling determination of diffusivity values (Figure 6a). In comparison to AOT diffusivity, each of the alternative solutes exhibits faster diffusion and specific values increase over the sequence: AOT < pHSA < SMO ≈ STO < OA. This order is somewhat surprising since diffusion is expected to be faster for smaller penetrants, and solutes’ relative molecular weights follow the decreasing sequence: STO > SMO ≈ AOT > OA (the grade of pHSA used does not have a published value). To investigate this observation, data were fit with eq 6 to yield *r*_*h*_ (via *D*_*0*_) and *k* (Figure 6b) – again, assuming ν = 1. The fitted values of solutes’ hopping frequencies and hydrodynamic radii help to rectify the mismatch between penetrant molecular weight and rate of diffusion—there is an inverse relationship between *k* and *r*_*h*_ (Figure 7).

**Figure 6:**
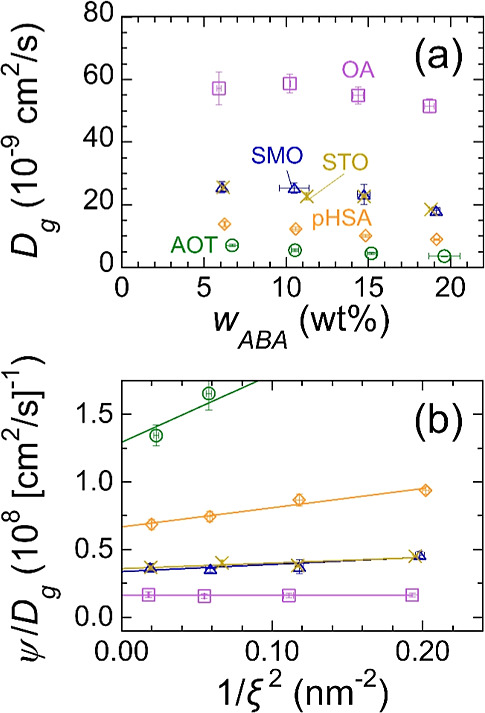
Solute diffusivity through organogels formulated with different solutes (see labels in (a)) presented in basic form (a) and linearized to the form of eq 6 (b) with corresponding parameters ψ and ξ from eqs 5 and 10, respectively. Solid lines in (b) are linear fits to each solute series. AOT data (initially presented in Figures 2b and 5) at the same copolymer concentrations are included for comparison.

**Figure 7:**
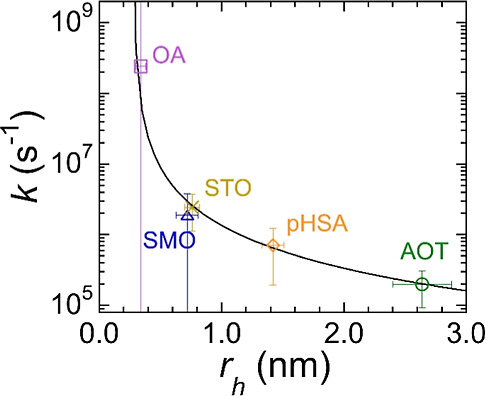
Correlation between hopping frequency and solute size for five examined solutes—labeled and color coded. The solid line is a guide to the eye.

The observed disconnect between solutes’ molecular weights and hydrodynamic radii is most likely linked to solute aggregation behavior. The aggregation of amphiphilic molecules in nonpolar solvent is less straightforward than aqueous solutions. Solutes dispersed in nonpolar solvents can exist as individual molecules (i.e., unimers), weak aggregate clusters, and/or conventional reverse micelles. Aggregate clusters generally have lower aggregation numbers than reverse micelles. Furthermore, they lack the well-defined structure associated with reverse micelles—a compact polar core surrounded by nonpolar tails/solvent.^32^ It is suspected that reverse micelle formation is nucleated by the presence of small amounts of water or other polar compounds because this drives molecules into more geometrically constrained aggregates. The absence of polar compounds greatly decreases the thermodynamic driving force for self-assembly explaining the lack of hierarchical structure in aggregate clusters. In the present MO-based gels, we hypothesize that OA exists as unimers; AOT primarily in reverse micelles; SMO and STO primarily in aggregate clusters; and pHSA as either unimers or in aggregate clusters.

Our hypothesis regarding solutes’ assembly behavior, or lack thereof, is based on their HLB numbers and SAXS analysis of gels comprising them. The HLB number of OA is 1.0 highlighting its strong lipophilicity. This combined with the fact that OA has been shown to suppress reverse micelle formation of other compounds (e.g., lecithin)^33^ suggests that it fully dissolves in MO. At the opposite end of the spectrum, AOT has an HLB number of 10.5 and is considered highly hygroscopic. Despite the thermal treatment used to prepare gels (i.e., 140 °C and 0.05 atm for 18–24 h), it is conceivable that a small amount of water, which is strongly bound to the charged sulfonate groups of AOT, is retained in the final gels. SMO and STO have moderate HLB numbers (4.3 and 1.8, respectively), and the HLB number of pHSA is likely in the range of 1–5 based on its general chemical structure. These values support the claim that SMO, STO, and pHSA may form weak associative interactions in MO but are not so great that the solutes would bind water throughout the gel preparation process.

It is further apparent from SAXS data that gels containing AOT are structurally unique from all other gels – they contain a broad shoulder at *q* = 1–3 nm^–1^ (Figures 8 and S4). To interpret this distinguishing feature, the noncopolymer aggregate term in eq 9 is incorporated into the structural model via

14 $$I\left(q\right)=\alpha_{ABA}P_{ABA}\left(q\right)S_{ABA}\left(q\right)+\alpha_{rm}P_{rm}\left(q\right)+bkg$$

where *P*_*ABA*_(*q*) and *S*_*ABA*_(*q*) retain the copolymer structural definitions described in eqs 7–8 and *P*_*rm*_(*q*) is the form factor of any reverse micelles present. The parameters α_*ABA*_ and α_*rm*_ are weighting factors for scattering from the copolymer domains and from solute reverse micelles, respectively, and are related to the concentration of each. Aggregate clusters are not expected to contribute a detectable level of scattered X-rays due to their low aggregation number and lack of well-defined structure.

**Figure 8:**
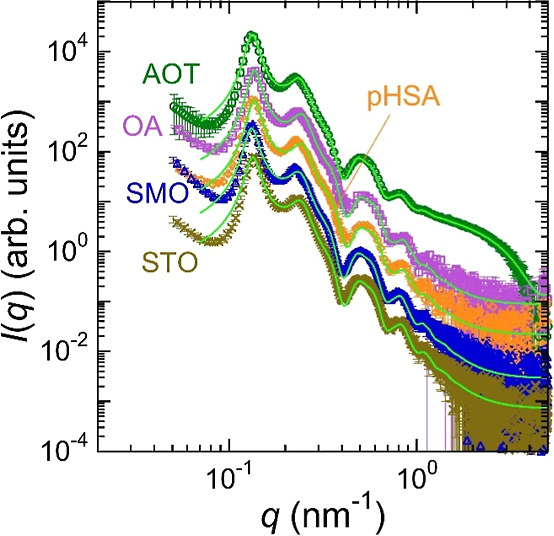
1D SAXS profiles for organogels prepared with ≈15 wt% S_22_EB_114_S_22_ and different solutes (labeled). Data are shifted vertically for clarity. Solid lines are fits to the data using eq 14 where α_*rm*_ = 0 for STO, SMO, pHSA, and OA (no reverse micelles present) or α_*rm*_ > 0 for AOT (includes reverse micelles).

Based on others’ work,^26^ we assume that AOT reverse micelles are spherical with a core radius, *r*_*rm*_, such that

15 $$P_{rm}\left(q\right)={\left(\frac{\sin\left(qr_{rm}\right)-qr_{rm}\cos\left(qr_{rm}\right)}{{\left(qr_{rm}\right)}^{3}}\right)}^{2}$$

The incorporation of this term captures the aforementioned shoulder in AOT-containing gels’ 1D SAXS profiles (Figures S3, S4, and S8), and the fitted value of *r*_*rm*_ remains constant across these formulations at 9.5 ± 0.1 Å. It was previously found that AOT reverse micelles dispersed in *n*-decane have a core radius of 9.4 Å further corroborating our results.^26^ Additionally, that study found a solvated AOT micelle radius of 19.8 Å, which is in the proximity of the hydrodynamic radius we extracted from the present diffusion experiments (*r*_*h*_ = 27 ± 2 Å). It would make sense that the hydrodynamic radius is greater than the solvated radius since the former is an effective size that could be enlarged by loosely bound solvent molecules surrounding each solvated reverse micelle. In the case of gels containing the other solutes employed, best fits yield α_*rm*_ = 0 indicating a lack of reverse micelles and, therefore, supporting the hypothesis of their presence as either unimers or aggregated in weak clusters.

#### Commentary on Solvent Quality

While we analyzed our data under the assumption that mineral oil is a theta solvent, it is likely that it actually falls on the upper end of the spectrum between good solvent and theta solvent (3/4 < ν < 1). Diffusivity data was reanalyzed under the other extreme – treatment of mineral oil as a good solvent (Figures S6–S8 and Table S11) – to determine whether decreasing ν affects any of our conclusions. Making the assumption of a good solvent translates to a considerable increase in mesh size for a given organogel formulation and results in a modest mesh size dependence on copolymer molecular weight (Figure 9).

**Figure 9:**
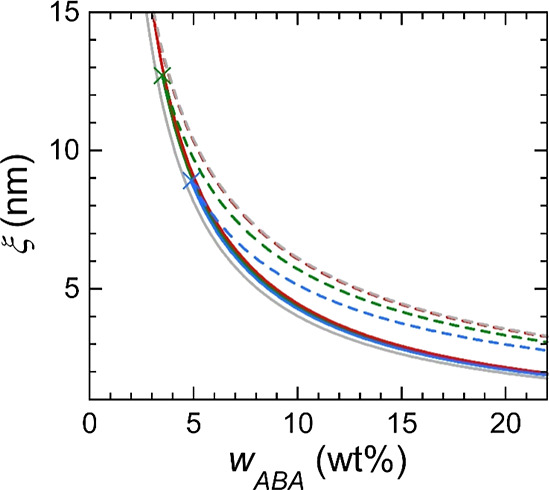
Matrix domain mesh size for organogels of varying copolymer concentration. Solid and dashed lines correspond to eq 10 where ν = 1 (theta solvent) and ν = 3/4 (good solvent), respectively, for each copolymer—blue: S_10_EB_55_S_10_, green: S_22_EB_114_S_22_, red: S_32_EB_170_S_32_, gray: S_61_EP_298_S_61_—and the x at the left end point of each reflects c*.

Through the same linearization fitting of data (Figures S6 and S7), we observe that there is minimal impact of solvent quality on the extracted *D*_*0*_ values, and consequently solute hydrodynamic radii (see Table S11 and Figure S8). Furthermore, *k* values remain within experimental uncertainty across the examined copolymer molecular weights (for a given solute) and follow the same qualitative trend as a function of solute *r*_*h*_. None of these findings change the conclusions drawn above. However, the magnitude of *k* values change with solvent quality. This is not surprising considering the growth of mesh size when ν → 3/4—solutes hop larger distances less frequently to accommodate the same rate of diffusion observed in Figure 2.

## Conclusion

The present study offers novel insights that should be taken into account when designing TDDSs based on block copolymer organogels. These insights were derived from experimental evaluation of solute diffusion through a variety of gel formulations including those with different copolymer concentrations and molecular weights, as well as gels comprising several unique solute compounds. Specifically, diffusivity values were extracted from solute retention curves and subsequently fitted using a composite model that considers the nanoscopic structure of triblock copolymer gels. Interpretation of data and corresponding model fits leads to several conclusions. First, solute diffusion depends on copolymer concentration because it leads to both increased polystyrene domain volume fraction and decreased matrix phase mesh size both of which reduce diffusion rate. For example, gels formulated at 5 wt% copolymer have ϕ_*S*_ ≈ 1% and ξ ≈ 8 nm translating to an AOT diffusivity of 7.5 × 10^–9^ cm^2^/s and those at 20 wt% copolymer have ϕ_*S*_ ≈ 5% and ξ ≈ 2 nm resulting in an AOT diffusivity of 3.5 × 10^–9^ cm^2^/s (assuming mineral oil is a theta solvent). Second, copolymer molecular weight has a minimal effect on solute diffusion when endblocks are near or above their entanglement molecular weight. The gels examined here, which maintain a constant polystyrene fraction across different molecular weights, exhibit roughly equivalent ϕ_*S*_, ξ, and AOT diffusivity. Finally, solute molecular weight is not a reliable reflection of penetrant size due to the complex molecular aggregation behavior in nonpolar solvents and, therefore, hydrodynamic radius should always be considered. It is important to acknowledge that these conclusions are solely focused on solute diffusivity. The design of effective TDDSs must also consider factors such as the strength of gel adhesion to skin and their flexibility, which complicates the design problem. However, we have previously provided some additional formulation strategies to decouple the transport and mechanical properties of these organogels.^34,35^ The combination of this and past work should enable effective bottom-up design of block copolymer organogels for TDDSs.

## Supplementary Material



## Notes

Copolymer concentration for all tested organogel formulations; thickness for all tested organogel formulations; representative FTIR spectra time series for each solute examined; solute retention curves for organogels varying in copolymer concentration and molecular weight; 1D SAXS profiles for organogels varying in copolymer concentration and molecular weight; 1D SAXS profiles for organogels containing different solutes; SAXS model fit parameters for all tested gel formulations; solute retention curves for organogels containing different solutes; side-by-side analysis of diffusivity data under theta solvent and good solvent treatment (PDF)

## †

Department of Chemical Engineering, Virginia Tech University, Blacksburg, VA 24060, USA

## Abbreviations

AOT: dioctyl sulfosuccinate sodium salt
FTIR: Fourier transfrom infrared spectroscopy
HLB: hydrophilic lipophilic balance
MO: aliphatic white mineral oil
OA: oleic acid
pHSA: poly­(hydroxystearic acid)
SAXS: small-angle X-ray scattering
SEBS: poly­(styrene-*b*-(ethylene-*co*-butylene)-*b*-styrene)
SEPS: poly­(styrene-*b*-(ethylene-*co*-propylene)-*b*-styrene)
SMO: sorbitan monooleate
STO: sorbitan trioleate
TDDS: transdermal drug delivery system
