# Supplementary material for: Solute Diffusion in Styrenic Triblock Copolymer Organogels
Source: ACS Appl Polym Mater. 2026 May 15;8(11):8568–76. doi: 10.1021/acsapm.6c01038 (PMC13270463; doi:10.1021/acsapm.6c01038)
Supplement: Supplementary file 1 [file ap6c01038_si_001.pdf]

Supporting Information for

Solute Diffusion in Styrenic Triblock Copolymer Organogels

Kenneth P. Mineart, Nicholas G. DeVita,† Ridwana Bashar*
Department of Chemical Engineering, Bucknell University, Lewisburg, PA 17837, USA

*corresponding author email: kpm007@bucknell.edu (K. P. Mineart)
†current address: Department of Chemical Engineering, Virginia Tech University, Blacksburg, VA 24060, USA

Table S1. Concentration of triblock copolymer in various gels following swelling – copolymer variation (diffusion probe = AOT).

$w_{ABA,0}$ (wt%)	w_{ABA} (wt%)			
	$S_{10}EB_{55}S_{10}$	$S_{22}EB_{114}S_{22}$	$S_{32}EB_{170}S_{32}$	$S_{61}EP_{298}S_{61}$
5.0	-	4.2 ± 0.1	-	2.7 ± 0.1
10.0	6.8 ± 0.1	6.7 ± 0.3	6.0 ± 0.1	4.6 ± 0.2
15.0	7.8 ± 0.6	8.7 ± 0.2	7.9 ± 0.1	6.0 ± 0.4
20.0	9.4 ± 0.1	10.5 ± 0.3	10.0 ± 0.1	7.5 ± 0.3
25.0	10.0 ± 0.6	13.0 ± 0.2	12.0 ± 0.2	9.4 ± 0.7
30.0	11.2 ± 0.1	15.2 ± 0.3	13.8 ± 0.3	10.9 ± 0.6
35.0	-	17.4 ± 0.2	-	-
40.0	-	19.6 ± 0.9	-	-

Table S2. Concentration of triblock copolymer in various gels following swelling – diffusion probe variation (copolymer = $S_{22}EB_{114}S_{22}$).

$w_{ABA,0}$ (wt%)	w_{ABA} (wt%)				
	AOT	OA	pHSA	SMO	STO
10.0	6.7 ± 0.3	5.9 ± 0.1	6.3 ± 0.1	6.1 ± 0.1	6.2 ± 0.1
20.0	10.5 ± 0.3	10.2 ± 0.1	10.6 ± 0.2	10.6 ± 0.4	11.3 ± 0.1
30.0	15.2 ± 0.3	14.4 ± 0.3	14.8 ± 0.2	14.8 ± 0.3	14.7 ± 0.2
40.0	19.6 ± 0.9	18.7 ± 0.3	19.2 ± 0.2	19.0 ± 0.2	18.8 ± 0.1

Table S3. Thickness of various gels following swelling – copolymer variation (solute = AOT).

$S_{10}EB_{55}S_{10}$		$S_{22}EB_{114}S_{22}$		$S_{32}EB_{170}S_{32}$		$S_{61}EP_{298}S_{61}$	
w_{ABA} (wt%)	2L (mm)	w_{ABA} (wt%)	2L (mm)	w_{ABA} (wt%)	2L (mm)	w_{ABA} (wt%)	2L (mm)
-	-	4.2 ± 0.1	1.59 ± 0.02	-	-	2.7 ± 0.1	1.70 ± 0.05
6.8 ± 0.1	1.53 ± 0.02	6.7 ± 0.3	1.72 ± 0.02	6.0 ± 0.1	1.82 ± 0.07	4.6 ± 0.2	1.85 ± 0.03
7.8 ± 0.6	1.66 ± 0.03	8.7 ± 0.2	1.75 ± 0.03	7.9 ± 0.1	1.87 ± 0.02	6.0 ± 0.4	2.06 ± 0.07
9.4 ± 0.1	1.70 ± 0.02	10.5 ± 0.3	1.81 ± 0.04	10.0 ± 0.1	2.00 ± 0.02	7.5 ± 0.3	2.10 ± 0.03
10.0 ± 0.6	1.78 ± 0.02	13.0 ± 0.2	1.84 ± 0.04	12.0 ± 0.2	2.09 ± 0.04	9.4 ± 0.7	2.31 ± 0.06
11.2 ± 0.1	1.85 ± 0.03	15.2 ± 0.3	1.89 ± 0.02	13.8 ± 0.3	2.10 ± 0.02	10.9 ± 0.6	2.53 ± 0.06
-	-	17.4 ± 0.2	2.03 ± 0.04	-	-	-	-
-	-	19.6 ± 0.9	2.11 ± 0.03	-	-	-	-

Table S4. Thickness of various gels following swelling – solute variation (copolymer = $S_{22}EB_{114}S_{22}$).

AOT		OA		pHSA	
w_{ABA} (wt%)	2L (mm)	w_{ABA} (wt%)	2L (mm)	w_{ABA} (wt%)	2L (mm)
6.7 ± 0.3	1.72 ± 0.02	5.9 ± 0.1	1.54 ± 0.05	6.3 ± 0.1	1.84 ± 0.03
10.5 ± 0.3	1.81 ± 0.04	10.2 ± 0.1	1.67 ± 0.03	10.6 ± 0.2	1.93 ± 0.01
15.2 ± 0.3	1.89 ± 0.02	14.4 ± 0.3	1.76 ± 0.01	14.8 ± 0.2	2.04 ± 0.08
19.6 ± 0.9	2.11 ± 0.03	18.7 ± 0.3	2.00 ± 0.05	19.2 ± 0.2	2.16 ± 0.03
**SMO**		**STO**			
w_{ABA} (wt%)	w_{ABA} (wt%)	w_{ABA} (wt%)	2L (mm)		
6.1 ± 0.1	1.74 ± 0.02	6.2 ± 0.1	1.77 ± 0.03		
10.6 ± 0.4	1.86 ± 0.03	11.3 ± 0.1	1.92 ± 0.03		
14.8 ± 0.3	2.08 ± 0.02	14.7 ± 0.2	2.07 ± 0.04		
19.0 ± 0.2	2.17 ± 0.02	18.8 ± 0.1	2.10 ± 0.02		

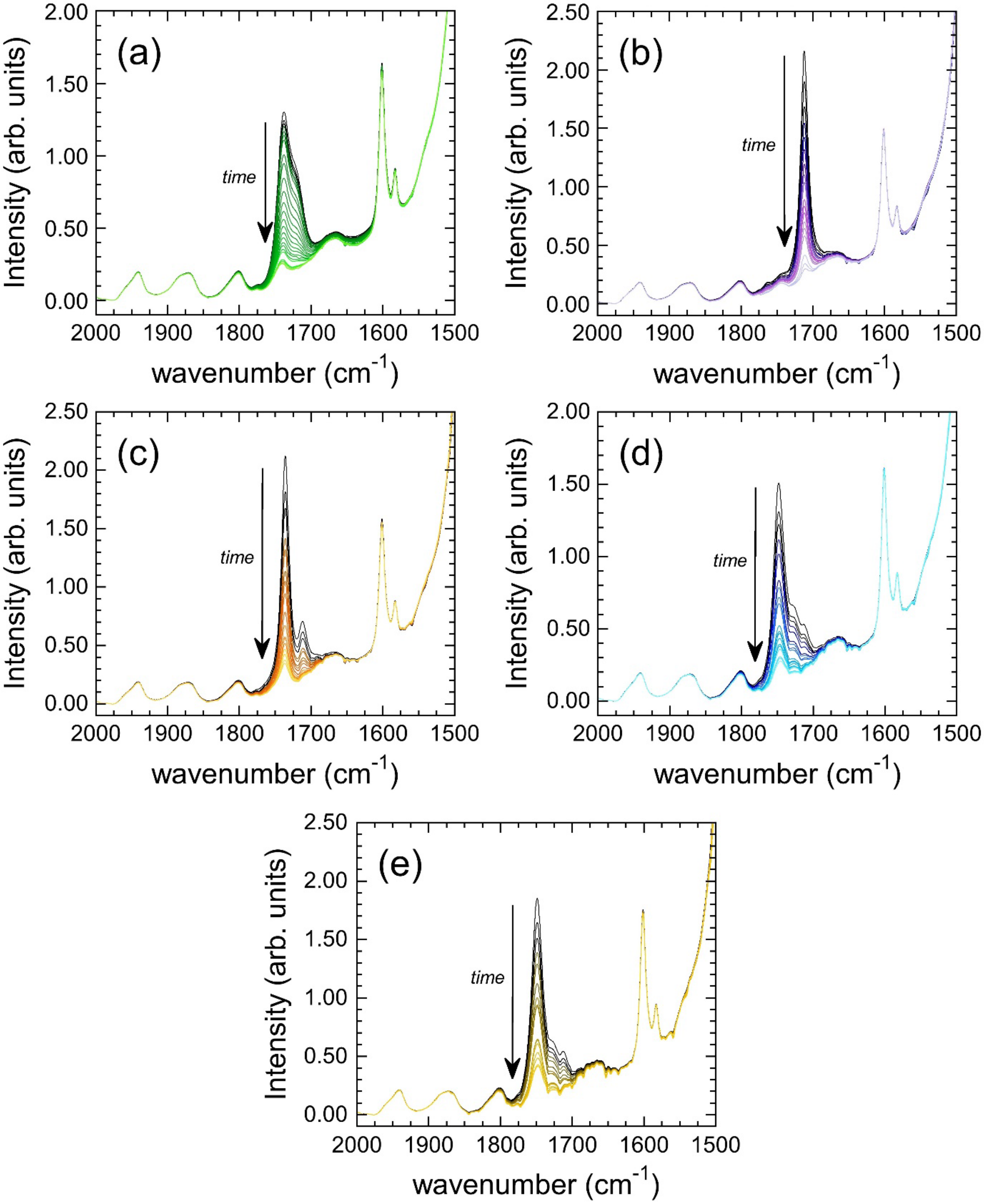

Figure S1. Representative FTIR spectra time series for organogels containing ≈11 wt% $S_{22}EB_{114}S_{22}$ (see Row 2 in Table S2) and different diffusion probes: (a) AOT, (b) OA, (c) pHSA, (d) SMO, and (e) STO. Note that only the primary ester/acid peak absorbance was used for subsequent analysis.

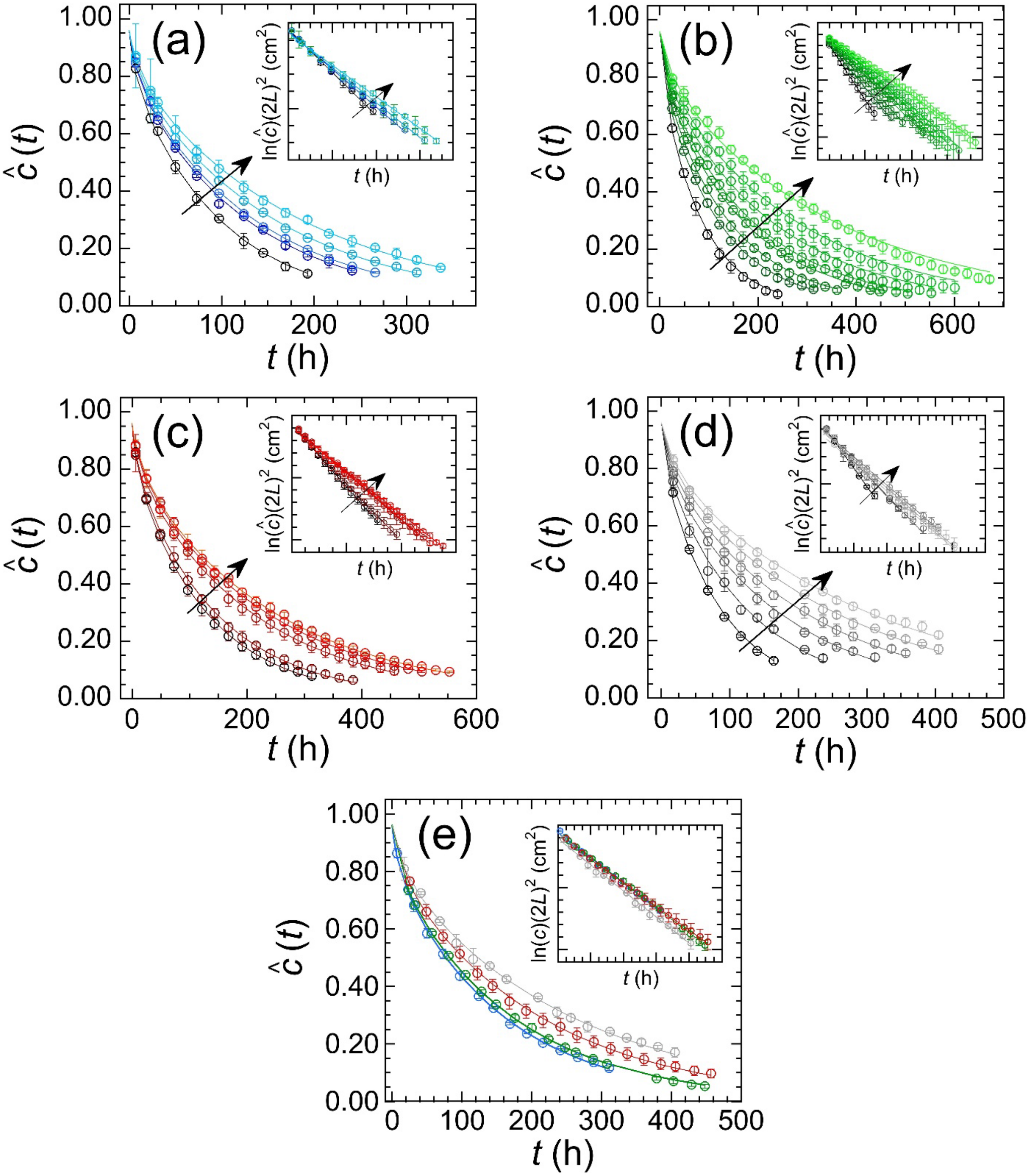

Figure S2. Solute retention curves for AOT diffusion through gels composed of (a) $S_{10}EB_{55}S_{10}$, (b) $S_{22}EB_{114}S_{22}$, (c) $S_{32}EB_{170}S_{32}$, and (d) $S_{61}EP_{298}S_{61}$ at increasing copolymer concentration (as indicated by the arrow in each figure), as well as a cross-copolymer comparison at roughly constant concentration (≈9.5-10.5 wt%). See Table S1 for specific concentrations. The insets show the same data plotted in quasi-linearized form. Solid lines reflect fitting with a truncated form of Equation 2 (in the article body): main figures = five terms, insets = first term only.

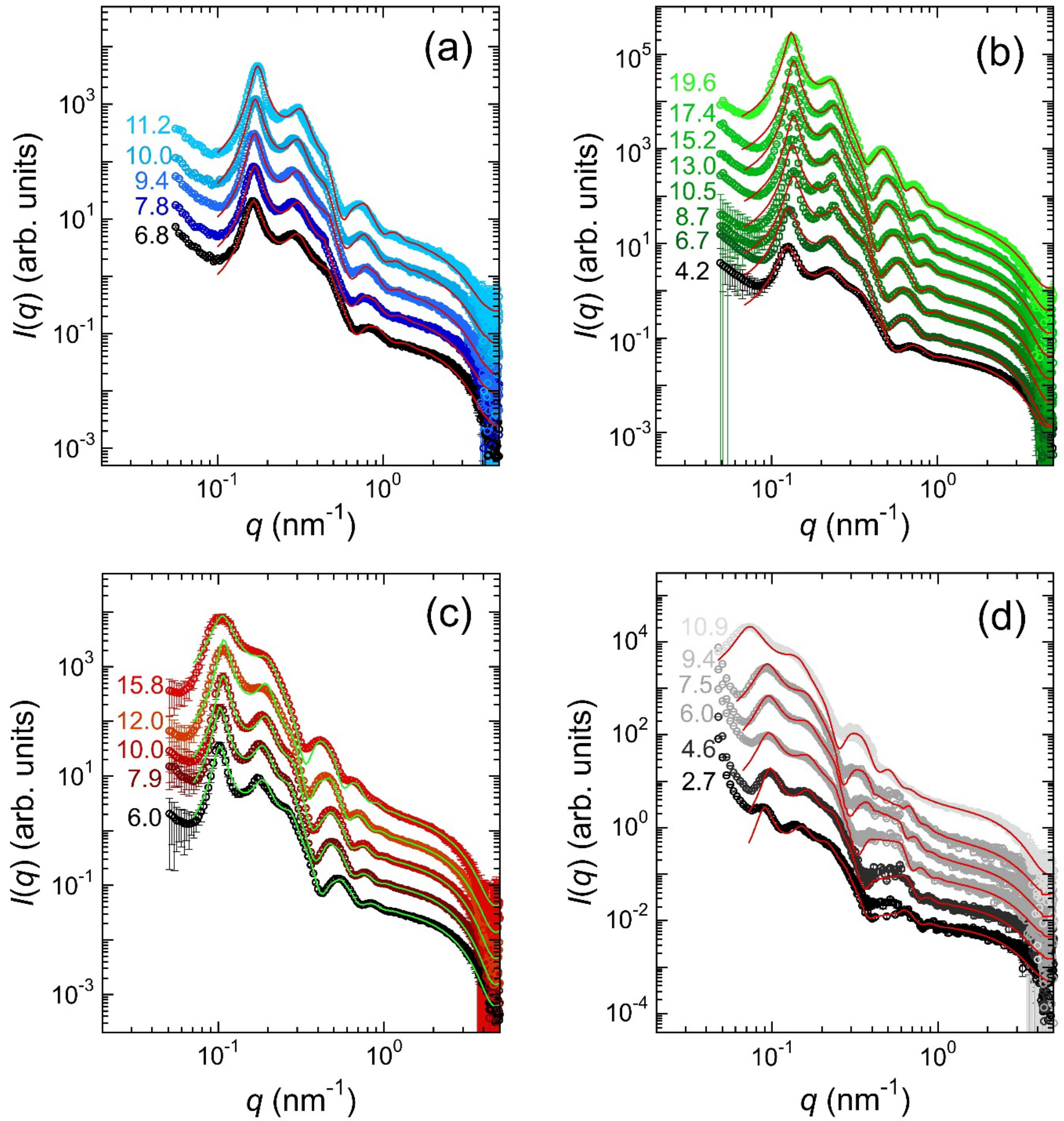

Figure S3. 1D SAXS profiles for organogels prepared with AOT as the solute and different copolymers – $S_{10}EB_{55}S_{10}$ (a), $S_{22}EB_{114}S_{22}$ (b), $S_{32}EB_{170}S_{32}$ (c), and $S_{61}EP_{298}S_{61}$ (d) – at several concentrations each (w_{ABA} (wt%) indicated with numeric labels). Data are shifted vertically for clarity. Solid lines are fits to the data using the copolymer model described in the text along with a spherical form factor to model the presence of AOT aggregates. The upturn in scattering at low-q arises from larger-scale heterogeneities not accounted for in the SAXS model. Therefore, fitting was performed starting from the initial minimum ($q \approx 0.07$-0.1 nm^{-1}).

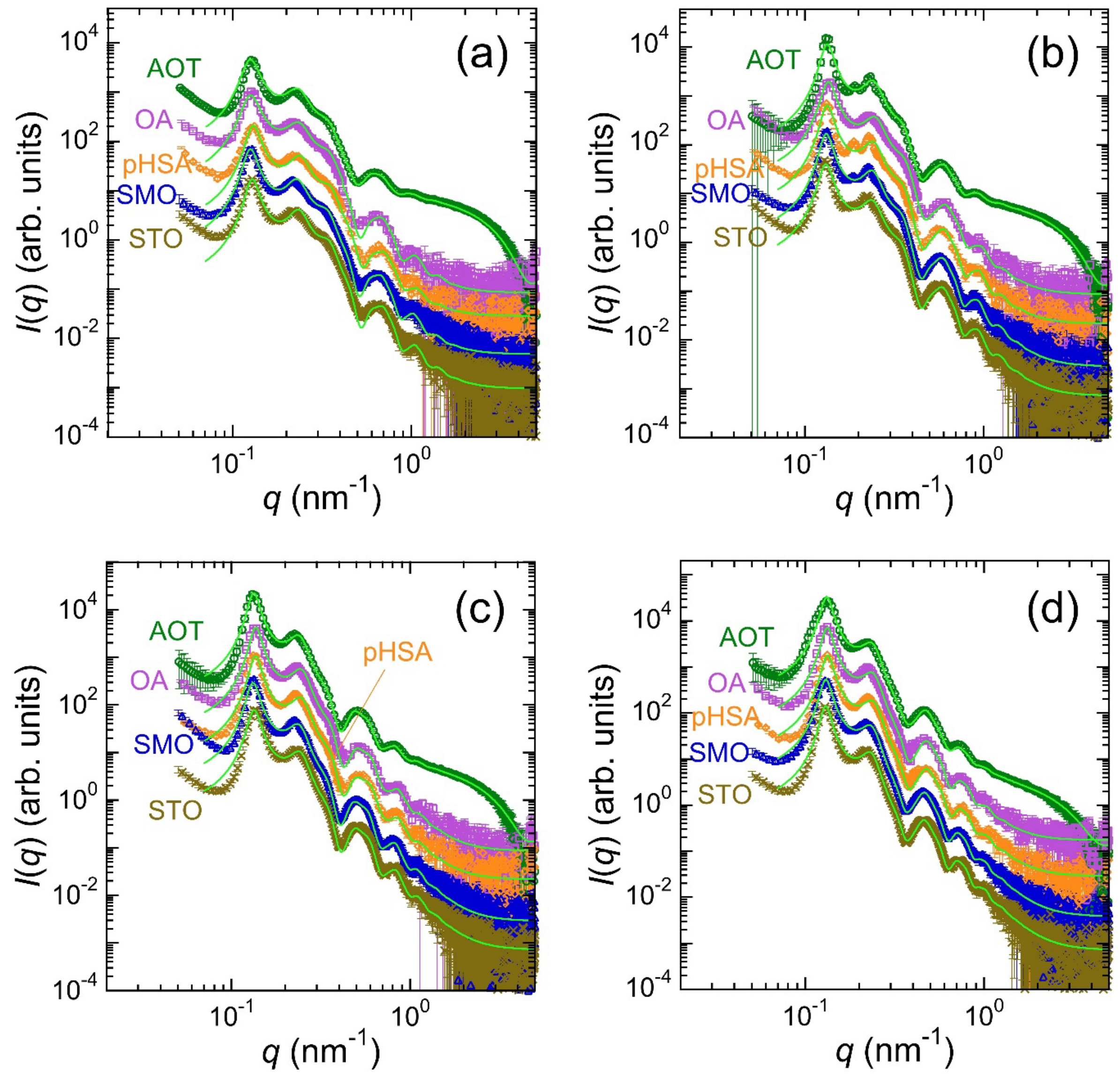

Figure S4. 1D SAXS profiles for organogels prepared with $S_{22}EB_{114}S_{22}$ as the copolymer at various concentrations – ≈6 wt% (a), ≈11 wt% (b), ≈15 wt% (c), and ≈19 wt% (d) – and different solutes (labeled in each). Data are shifted vertically for clarity. Solid lines are fits to the data using the copolymer model described in the text along with a spherical form factor to model the presence of AOT aggregates (only in the case of organogels containing AOT). The upturn in scattering at low-q arises from larger-scale heterogeneities not accounted for in the SAXS model. Therefore, fitting was performed starting from the initial minimum ($q \approx 0.07$-0.1 nm^{-1}).

Table S5. Polystyrene endblock domain radius (r_S) of various gels based on fitting of SAXS data – copolymer variation (solute = AOT).

$S_{22}EB_{114}S_{22}$		$S_{22}EB_{114}S_{22}$		$S_{61}EP_{298}S_{61}$		$S_{61}EP_{298}S_{61}$	
w_{ABA} **(wt%)**	r_S **(nm)**	w_{ABA} **(wt%)**	r_S **(-)**	w_{ABA} **(wt%)**	r_S **(-)**	w_{ABA} **(wt%)**	r_S **(-)**
-	-	4.2 ± 0.1	7.8	-	-	2.7 ± 0.1	11.8
6.8 ± 0.1	6.6	6.7 ± 0.3	8.9	6.0 ± 0.1	10.5	4.6 ± 0.2	12.7
7.8 ± 0.6	6.9	8.7 ± 0.2	8.9	7.9 ± 0.1	11.6	6.0 ± 0.4	14.2
9.4 ± 0.1	7.1	10.5 ± 0.3	9.7	10.0 ± 0.1	11.8	7.5 ± 0.3	15.8
10.0 ± 0.6	7.4	13.0 ± 0.2	10.2	12.0 ± 0.2	12.5	9.4 ± 0.7	17.4
11.2 ± 0.1	7.5	15.2 ± 0.3	11.0	13.8 ± 0.3	13.2	10.9 ± 0.6	17.5
-	-	17.4 ± 0.2	11.1	-	-	-	-
-	-	19.6 ± 0.9	12.0	-	-	-	-

Table S6. Polystyrene endblock domain radius (r_S) of various gels based on fitting of SAXS data – solute variation (copolymer = $S_{22}EB_{114}S_{22}$).

AOT		OA		pHSA	
w_{ABA} **(wt%)**	r_S **(nm)**	w_{ABA} **(wt%)**	r_S **(nm)**	w_{ABA} **(wt%)**	r_S **(nm)**
6.7 ± 0.3	8.9	5.9 ± 0.1	8.4	6.3 ± 0.1	8.3
10.5 ± 0.3	9.7	10.2 ± 0.1	9.3	10.6 ± 0.2	9.6
15.2 ± 0.3	11.0	14.4 ± 0.3	10.5	14.8 ± 0.2	10.5
19.6 ± 0.9	12.0	18.7 ± 0.3	11.8	19.2 ± 0.2	11.8

SMO		STO	
w_{ABA} **(wt%)**	r_S **(nm)**	w_{ABA} **(wt%)**	r_S **(nm)**
6.1 ± 0.1	8.7	6.2 ± 0.1	8.5
10.6 ± 0.4	9.8	11.3 ± 0.1	9.8
14.8 ± 0.3	11.0	14.7 ± 0.2	10.9
19.0 ± 0.2	12.2	18.8 ± 0.1	12.2

Table S7. Hard sphere radius (r_{hs}) and volume fraction (ϕ_{hs}) of various gels based on fitting of SAXS data – copolymer variation (solute = AOT).

$S_{10}EB_{55}S_{10}$			$S_{22}EB_{114}S_{22}$			$S_{32}EB_{170}S_{32}$			$S_{61}EP_{298}S_{61}$		
w_{ABA} (wt%)	r_{hs} (nm)	ϕ_{hs} (-)	w_{ABA} (wt%)	r_{hs} (nm)	ϕ_{hs} (-)	w_{ABA} (wt%)	r_{hs} (nm)	ϕ_{hs} (-)	w_{ABA} (wt%)	r_{hs} (nm)	ϕ_{hs} (-)
-	-	-	4.2 ± 0.1	26.9	0.42	-	-	-	2.7 ± 0.1	48.0	0.41
6.8 ± 0.1	20.6	0.45	6.7 ± 0.3	27.0	0.45	6.0 ± 0.1	34.0	0.49	4.6 ± 0.2	43.5	0.41
7.8 ± 0.6	20.5	0.47	8.7 ± 0.2	25.5	0.48	7.9 ± 0.1	34.0	0.50	6.0 ± 0.4	43.0	0.35
9.4 ± 0.1	20.7	0.48	10.5 ± 0.3	26.2	0.50	10.0 ± 0.1	32.8	0.51	7.5 ± 0.3	43.0	0.34
10.0 ± 0.6	20.5	0.48	13.0 ± 0.2	25.9	0.51	12.0 ± 0.2	32.5	0.51	9.4 ± 0.7	43.5	0.33
11.2 ± 0.1	19.8	0.48	15.2 ± 0.3	26.4	0.51	13.8 ± 0.3	32.0	0.43	10.9 ± 0.6	43.5	0.35
-	-	-	17.4 ± 0.2	26.0	0.52	-	-	-	-	-	-
-	-	-	19.6 ± 0.9	26.7	0.51	-	-	-	-	-	-

Table S8. Hard sphere radius (r_{hs}) and volume fraction (ϕ_{hs}) of various gels based on fitting of SAXS data – solute variation (copolymer = $S_{22}EB_{114}S_{22}$).

AOT			OA			pHSA		
w_{ABA} (wt%)	r_{hs} (nm)	ϕ_{hs} (-)	w_{ABA} (wt%)	r_{hs} (nm)	ϕ_{hs} (-)	w_{ABA} (wt%)	r_{hs} (nm)	ϕ_{hs} (-)
6.7 ± 0.3	27.0	0.45	5.9 ± 0.1	26.8	0.44	6.3 ± 0.1	26.5	0.48
10.5 ± 0.3	26.2	0.50	10.2 ± 0.1	25.5	0.48	10.6 ± 0.2	26.2	0.50
15.2 ± 0.3	26.4	0.51	14.4 ± 0.3	25.7	0.51	14.8 ± 0.2	25.8	0.51
19.6 ± 0.9	26.7	0.51	18.7 ± 0.3	26.5	0.51	19.2 ± 0.2	26.4	0.51

SMO			STO		
w_{ABA} (wt%)	r_{hs} (nm)	ϕ_{hs} (-)	w_{ABA} (wt%)	r_{hs} (nm)	ϕ_{hs} (-)
6.1 ± 0.1	27.5	0.48	6.2 ± 0.1	27.0	0.49
10.6 ± 0.4	26.5	0.50	11.3 ± 0.1	26.5	0.49
14.8 ± 0.3	26.5	0.50	14.7 ± 0.2	26.1	0.50
19.0 ± 0.2	27.0	0.51	18.8 ± 0.1	27.2	0.52

Table S9. Polystyrene domain volume fraction (ϕ_S) of various gels based on fitting of SAXS data, as well as gel heterogeneity 'correction factor' (ψ) – copolymer variation (solute = AOT).

$S_{10}EB_{55}S_{10}$			$S_{22}EB_{114}S_{22}$			$S_{32}EB_{170}S_{32}$			$S_{61}EP_{298}S_{61}$		
w_{ABA} (wt%)	ϕ_S (-)	ψ (-)	w_{ABA} (wt%)	ϕ_S (-)	ψ (-))	w_{ABA} (wt%)	ϕ_S (-)	ψ (-)	w_{ABA} (wt%)	ϕ_S (-)	ψ (-)
-	-	-	4.2 ± 0.1	0.010	0.96	-	-	-	2.7 ± 0.1	0.006	0.97
6.8 ± 0.1	0.015	0.94	6.7 ± 0.3	0.016	0.94	6.0 ± 0.1	0.014	0.94	4.6 ± 0.2	0.010	0.96
7.8 ± 0.6	0.018	0.93	8.7 ± 0.2	0.020	0.92	7.9 ± 0.1	0.020	0.92	6.0 ± 0.4	0.013	0.94
9.4 ± 0.1	0.019	0.92	10.5 ± 0.3	0.025	0.91	10.0 ± 0.1	0.024	0.90	7.5 ± 0.3	0.017	0.93
10.0 ± 0.6	0.023	0.91	13.0 ± 0.2	0.031	0.89	12.0 ± 0.2	0.029	0.88	9.4 ± 0.7	0.021	0.91
11.2 ± 0.1	0.026	0.90	15.2 ± 0.3	0.037	0.87	13.8 ± 0.3	0.030	0.87	10.9 ± 0.6	0.023	0.90
-	-	-	17.4 ± 0.2	0.040	0.85	-	-	-	-	-	-
-	-	-	19.6 ± 0.9	0.046	0.84	-	-	-	-	-	-

Table S10. Polystyrene domain volume fraction (ϕ_S) of various gels based on fitting of SAXS data, as well as gel heterogeneity 'correction factor' (ψ) – solute variation (copolymer = $S_{22}EB_{114}S_{22}$).

AOT			OA			pHSA		
w_{ABA} (wt%)	ϕ_S (-)	ψ (-)	w_{ABA} (wt%)	ϕ_S (-)	ψ (-)	w_{ABA} (wt%)	ϕ_S (-)	ψ (-)
6.7 ± 0.3	0.016	0.94	5.9 ± 0.1	0.014	0.95	6.3 ± 0.1	0.015	0.94
10.5 ± 0.3	0.025	0.91	10.2 ± 0.1	0.023	0.91	10.6 ± 0.2	0.025	0.91
15.2 ± 0.3	0.037	0.87	14.4 ± 0.3	0.033	0.88	14.8 ± 0.2	0.034	0.87
19.6 ± 0.9	0.046	0.84	18.7 ± 0.3	0.043	0.84	19.2 ± 0.2	0.046	0.84

SMO			STO		
w_{ABA} (wt%)	ϕ_S (-)	ψ (-)	w_{ABA} (wt%)	ϕ_S (-)	ψ (-)
6.1 ± 0.1	0.015	0.95	6.2 ± 0.1	0.015	0.95
10.6 ± 0.4	0.025	0.91	11.3 ± 0.1	0.025	0.90
14.8 ± 0.3	0.036	0.87	14.7 ± 0.2	0.036	0.87
19.0 ± 0.2	0.047	0.84	18.8 ± 0.1	0.047	0.84

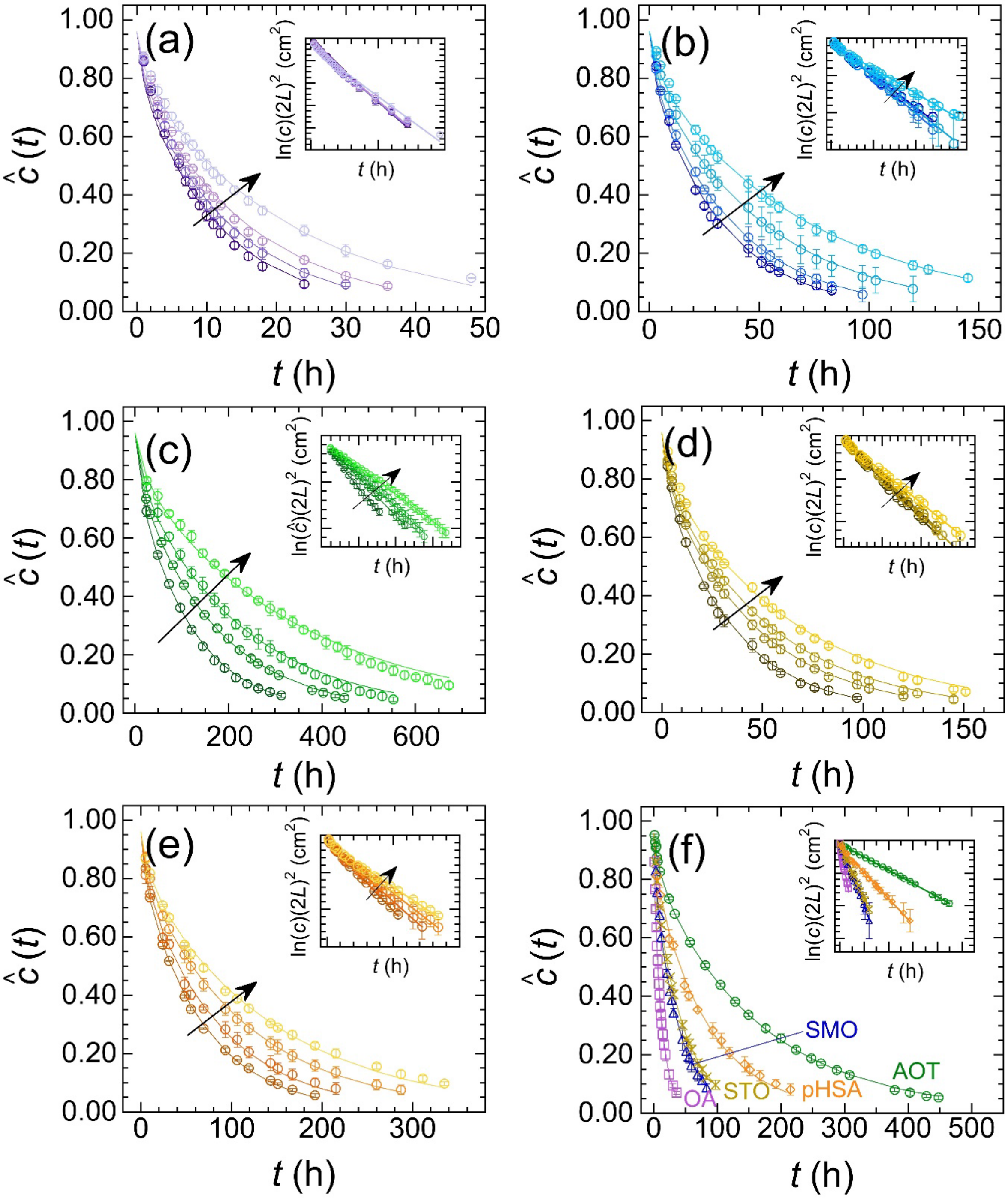

Figure S5. Solute retention curves for gels composed of $S_{22}EB_{114}S_{22}$ at increasing copolymer concentration (as indicated by the arrow in each figure) and with different solutes: (a) OA, (b) SMO, (c) AOT, (d) STO, and (e) pHSA, as well as a cross-solute comparison at roughly constant concentration (≈11 wt%). See Table S2 for specific concentrations. The insets show the same data plotted in quasi-linearized form. Solid lines reflect fitting with a truncated form of Equation 2 (in the article body): main figures = five terms, insets = first term only.

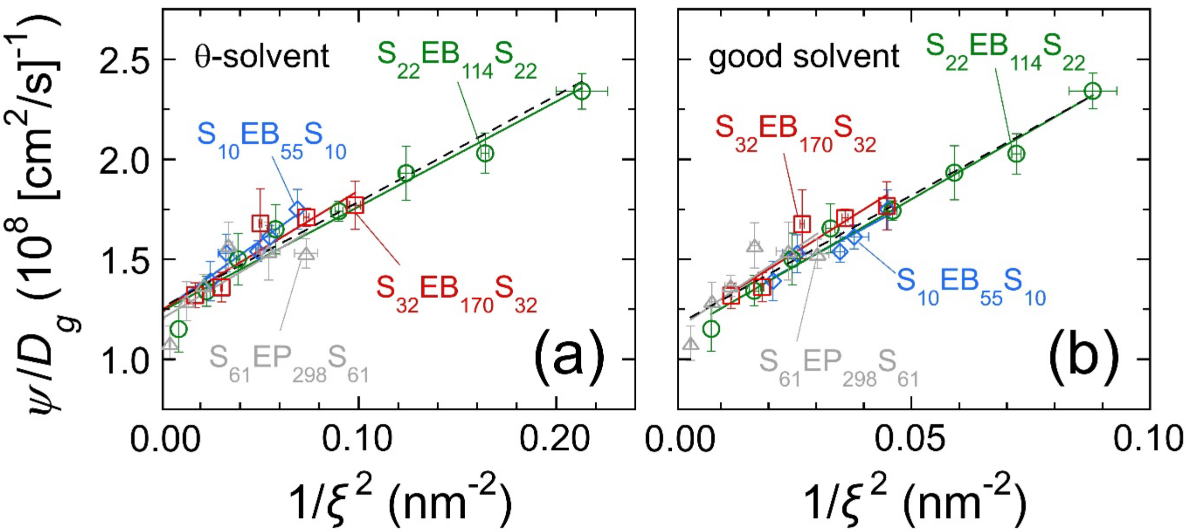

Figure S6. Solute diffusivity values for AOT diffusion through organogels formulated with different copolymer concentrations and molecular weights (labeled) presented in the linearized form of Equation 6 under treatment of mineral oil as a θ-solvent (a) and a good solvent (b). Solid lines are linear fits to each copolymer series, and the dashed line is an overall fit to all data.

Table S11. Parameter values extracted from fitting solute diffusivity data in Figure 5 across different copolymer identities and under treatment of mineral oil as a θ-solvent (left) and good solvent (right) along with resultant values from fitting all data together in each case.

copolymer	**D_0 (10^{-9} cm²/s)**	**k (10^5 s⁻¹)**
$S_{10}EB_{55}S_{10}$	8.06 ± 0.67	1.43 ± 0.51
$S_{22}EB_{114}S_{22}$	8.06 ± 0.74	1.91 ± 0.25
$S_{32}EB_{170}S_{32}$	8.03 ± 0.84	1.69 ± 0.87
$S_{61}EP_{298}S_{61}$	8.34 ± 0.90	1.71 ± 1.07
all data	*7.95 ± 0.63*	*1.89 ± 1.01*
	r_h = 2.7 ± 0.2 nm	

copolymer	**D_0 (10^{-9} cm²/s)**	**k (10^5 s⁻¹)**
$S_{10}EB_{55}S_{10}$	8.74 ± 0.89	0.79 ± 0.23
$S_{22}EB_{114}S_{22}$	8.94 ± 0.88	0.73 ± 0.44
$S_{32}EB_{170}S_{32}$	8.70 ± 1.04	0.67 ± 0.23
$S_{61}EP_{298}S_{61}$	8.79 ± 1.03	0.62 ± 0.31
all data	*8.60 ± 0.73*	*0.76 ± 0.59*
	r_h = 2.5 ± 0.2 nm	

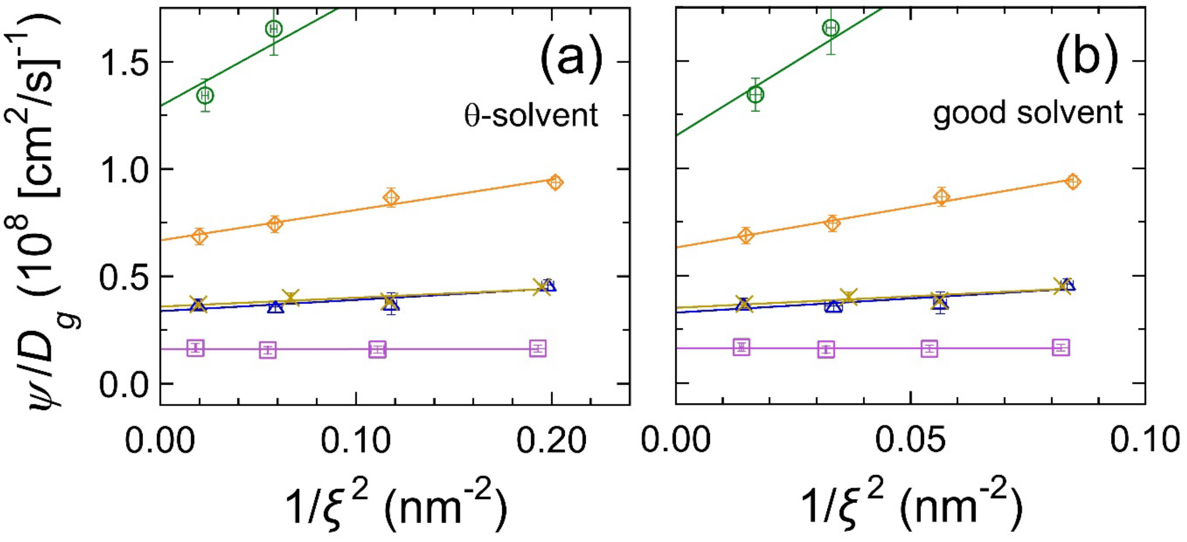

Figure S7. Solute diffusivity through organogels formulated with different solutes presented in the linearized form of Equation 6 under treatment of mineral oil as a θ-solvent (a) and a good solvent (b). Solid lines are linear fits to each solute series. AOT data (fully presented in Figure S7) at the same copolymer concentrations are included for comparison.

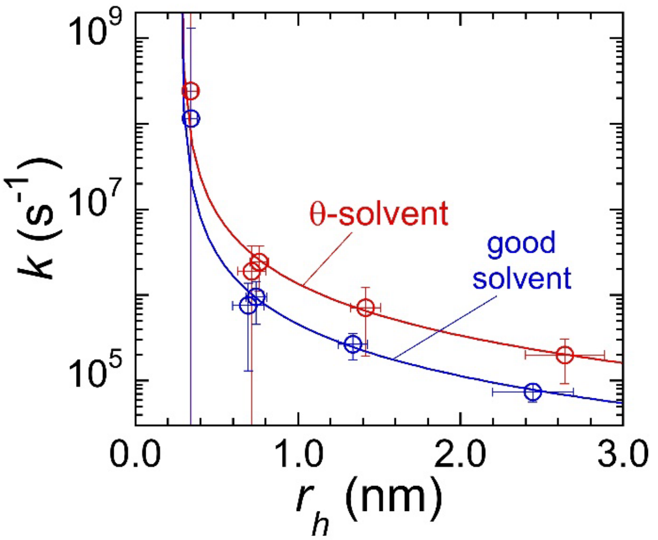

Figure S8. Correlation between hopping frequency and solute size for five examined solutes assuming θ-solvent and good solvent (labeled and color-coded). Solid lines are guides to the eye.
